# Cutaneous Lymphomas and Lymphoproliferative Disorders Associated With SARS‐CoV‐2 Vaccination: A Systematic Review

**DOI:** 10.1155/jskc/4893577

**Published:** 2026-05-28

**Authors:** Martina Mussi, Corrado Zengarini, Alberto Corrà, Tullio Brunetti, Alessio Natale, Michelangelo La Placa, Bianca Maria Piraccini, Alba Guglielmo, Alessandro Pileri

**Affiliations:** ^1^ Department of Medical and Surgical Sciences, University of Bologna, Bologna, Italy, unibo.it; ^2^ Dermatology Unit, IRCCS Azienda Ospedaliero-Universitaria di Bologna, Bologna, Italy; ^3^ Dermatology Unit, Ospedale San Bortolo, Vicenza, Italy, ulssvicenza.it; ^4^ Department of Medical Sciences, Section of Hematology and Rheumatology, University of Ferrara, Ferrara, Italy, unife.it

**Keywords:** CD30, lymphoma, skin, T cells, vaccination

## Abstract

**Background:**

Cutaneous lymphomas (CLs) are rare neoplastic skin disorders, primarily of T‐cell origin. Since the widespread rollout of SARS‐CoV‐2 vaccines, several cases of CLs and non‐neoplastic lymphoproliferative disorders (nn‐LPDs) temporally associated with vaccination have been reported, raising concerns about a potential immunologic link.

**Objective:**

To systematically review the literature on cases of CLs and related nn‐LPDs occurring after COVID‐19 vaccination, focusing on clinical features, subtype distribution, latency to onset, and proposed pathophysiological mechanisms.

**Methods:**

A systematic review was conducted according to PRISMA guidelines. Case reports and series describing new‐onset or relapsed CLs or nn‐LPDs temporally following SARS‐CoV‐2 vaccination were included. Demographic, clinical, histological, therapeutic and temporal data were extracted and analysed.

**Results:**

Fifteen manuscripts encompassing 35 cases met the inclusion criteria. Eighteen (51.4%) were histologically confirmed CLs, most commonly lymphomatoid papulosis (*n* = 9), followed by Sézary syndrome (*n* = 3) and mycosis fungoides (*n* = 2). The remaining 17 cases (48.6%) were classified as nn‐LPDs, including cutaneous lymphoid hyperplasia, lymphomatoid reactions and CD4+ small/medium T‐cell lymphoproliferative disorders. CD30 positivity was noted in 76.2% of the cases with available immunophenotyping. Most patients (80%) received the BNT162b2 (Pfizer–BioNTech) vaccine. In the 17 new‐onset CLs, time to onset ranged from 3 to 42 days, with most cases clustering within 14 days.

**Conclusions:**

Most of the cases were low‐grade T‐cell CLs and nn‐LPDs. Although a causal relationship cannot be established, the short latency observed in many new‐onset cases raises the possibility that some patients may have harboured an undiagnosed disease, with vaccination acting as a trigger for clinical manifestation or for raised awareness. These findings support indeed the need for continued pharmacovigilance among clinicians, especially in patients with prior or latent lymphoproliferative conditions. Nevertheless, these extremely rare and indolent events should not alter the overall favourable risk–benefit profile of COVID‐19 vaccination.

## 1. Introduction

The rapid deployment of COVID‐19 vaccines has been a pivotal achievement in controlling the global pandemic, significantly reducing the severity of disease and mortality. However, as with any large‐scale medical intervention, widespread vaccination has led to the emergence of rare adverse events, necessitating further investigation. Among these, primary cutaneous lymphoid proliferations (PCLPs), which include cutaneous lymphomas (CLs) and non‐neoplastic lymphoproliferative disorders (nn‐LPDs)—a heterogeneous group of skin‐dominant lymphoproliferative disorders—have been reported in temporal association with vaccination, raising questions about the potential immunological mechanisms involved [1–4]. Regardless of their platform, all vaccines are designed to stimulate the immune system to confer protection against infectious diseases. While generally well tolerated, vaccination can lead to various unintended immune responses, including allergic, inflammatory and systemic adverse events.

Hypersensitivity reactions, including anaphylaxis and delayed cutaneous eruptions, have been reported following COVID‐19 vaccination, particularly with mRNA vaccines due to excipients, such as polyethylene glycol. Inflammatory and autoimmune manifestations have also emerged, with cases of myocarditis, immune thrombocytopenia, vasculitis and vaccine‐induced thrombotic thrombocytopenia associated with both mRNA and adenoviral vector‐based vaccines [5–8].

Additionally, vaccine‐driven cytokine release has been implicated in systemic symptoms, such as fever, myalgia and fatigue, while rare cases of multisystem inflammatory syndrome and de novo autoimmune conditions have also been documented [9, 10]. Within the dermatologic spectrum, a broad array of cutaneous adverse events has been observed postvaccination, ranging from localized erythema and induration at the injection site to widespread morbilliform eruptions, urticaria, livedo reticularis and erythema multiforme‐like presentations [11–13]. More severe reactions, including Stevens–Johnson syndrome, toxic epidermal necrolysis and autoimmune bullous dermatoses, have been described, albeit rarely [14–16]. Moreover, vaccination appears to influence pre‐existing dermatologic conditions, with exacerbations reported in psoriasis, atopic dermatitis, lichen planus and chronic urticaria [17–21].

The ability of SARS‐CoV‐2 vaccines—particularly mRNA‐based platforms—to elicit potent immune responses has raised discussion about how transient immune activation might interact with latent or indolent lymphoproliferative conditions, in susceptible individuals. CLs represent a heterogeneous group of extranodal non‐Hodgkin lymphomas, primarily comprising cutaneous T‐cell lymphomas (CTCLs), with cutaneous B‐cell lymphomas (CBCLs) accounting for a smaller fraction [22–24]. Among CTCLs, lymphomatoid papulosis (LyP) and primary cutaneous CD30+ lymphoproliferative disorders often follow an indolent clinical course. In contrast, others—such as Sézary syndrome (SS) and primary cutaneous *γ*/*δ* T‐cell lymphoma—are characterized by aggressive behaviour and poor prognosis [25–27]. Mycosis fungoides (MF), the most common CTCL subtype, spans a broad clinical spectrum, ranging from slowly progressive early stages to more advanced, treatment‐refractory forms [28–31].

The pathogenesis of CLs is multifactorial, involving chronic antigenic stimulation, genetic susceptibility and immune dysregulation within the skin‐homing T‐cell compartment [32–34]. In this context, vaccine‐induced immune activation may precipitate unmasking subclinical disease or exacerbate pre‐existing lymphoproliferative clones, particularly in immunologically predisposed individuals [35], through several nonmutually exclusive mechanisms. First, innate priming and type I interferon burst, as mRNA–lipid nanoparticles and other platform components, have the capacity of activating TLR7/8 and related sensors, inducing type I IFN and an early cytokine pulse (e.g., IL‐6 and TNF‐α) that amplifies antigen presentation and T‐cell costimulation. In predisposed individuals, this ‘danger signal’ could unmask subclinical clones by increasing their activation/survival signals.

Also, bystander T‐cell activation and homeostatic proliferation could act in the pathogenic mechanism, as the vaccine‐driven cytokine milieu (IL‐2, IL‐7 and IL‐15) supports bystander expansion of memory/effector skin‐tropic T cells (CLA+, CCR4/CCR10+). If a pre‐existing cutaneous clone is present, it may expand transiently, translating into new lesions or relapse without implying de novo oncogenesis.

Moreover, CD30 pathway and NF‐κB signalling in indolent CTCLs, as in CD30‐positive disorders (LyP/pcALCL spectrum), inflammatory cytokines and costimulatory inputs, can upmodulate CD30 and downstream pathways (NF‐κB, JAK/STAT), fostering short‐latency flares that typically recede as systemic activation wanes—consistent with a ‘trigger rather than initiator’ model.

Finally, trafficking and microenvironmental cues, as vaccination reshapes chemokine gradients and adhesion dynamics, potentially increase skin homing of activated T cells and amplify local cytokine cross‐talk with keratinocytes and dendritic cells; this favours temporary clinical expression of disease in patients with pre‐existing clonal T‐cell populations.

This review critically examines the current evidence linking COVID‐19 vaccination to the onset or relapse of PCLPs, with a focus on CTCLs, discusses proposed immunopathological mechanisms and explores implications for clinical surveillance and vaccine safety. Clarifying whether these associations reflect causal mechanisms or temporal coincidences is essential to support both informed patient management and continued public confidence in vaccination strategies.

## 2. Material and Methods

### 2.1. Search Strategy and Study Selection

This review followed the Preferred Reporting Items for Systematic Reviews and Meta‐Analyses (PRISMA) guidelines [36]. A comprehensive search was performed in PubMed/MEDLINE, Scopus and Web of Science databases using the following Boolean keyword combinations:

(“cutaneous lymphoma” OR “lymphoproliferative disorders”) AND (“COVID‐19 vaccination” OR “SARS‐CoV‐2 vaccination”). The last search was completed on 06 March 2026. No initial restrictions were applied regarding publication language, year, journal type or geographical origin. Filters were applied after full‐text screening.

Articles were selected by two reviewers independently, who screened titles and/or abstracts and then full texts against prespecified inclusion/exclusion criteria. Disagreements were resolved by discussion or, when needed, by a third reviewer. They elected articles if they reported: cases of new‐onset or CLs recurrences exacerbated by COVID‐19 vaccination; included definitive diagnoses based on histopathology; detailed patient demographics, vaccine type and clinical outcomes. We did not restrict by study design (case reports/series, observational studies, trials) with the exception of reviews and editorials.

Exclusion criteria were as follows: (1) review articles; (2) articles not written in English; (3) patients under 18 years; (4) lymphomas not primarily cutaneous; (5) cases lacking histopathological confirmation; and (6) duplicate reports.

### 2.2. Data Extraction and Synthesis

The following information was collected: first author, journal of publication, article title and year of publication. Data extracted included: (a) author, title, journal, year of publication; (b) patient demographics (age at clinical onset/relapse temporally associated with vaccination and gender); (c) type of lymphoma and subtype; (d) vaccine type and timing; (e) latency period (days to onset); (f) clinical manifestations; (g) therapeutic approach; and (h) outcomes (resolution, relapse, death and cause of death).

## 3. Results

**FIGURE 1:**
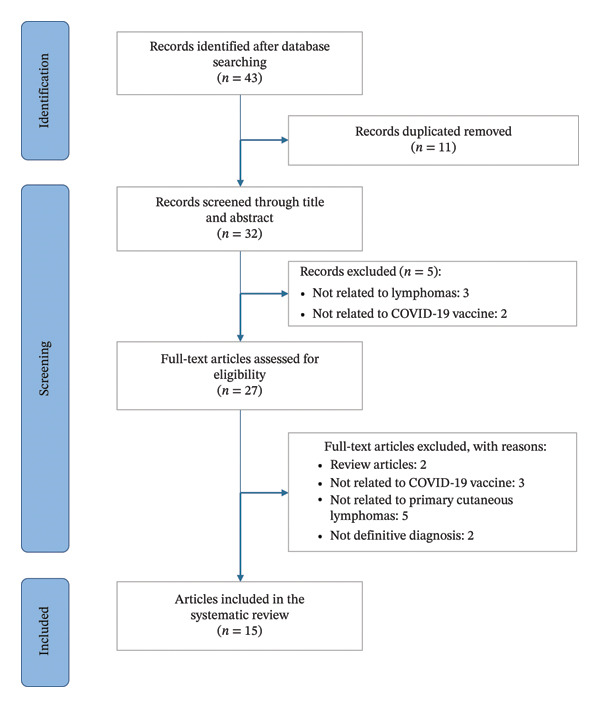
Flow diagram of the study according to PRISMA.

A total of 35 cases of cutaneous lymphoproliferative disorders temporally associated with COVID‐19 vaccination were identified across 32 publications, after removing 11 duplicates from an initial set of 43 articles. After title and abstract screening and full‐text assessment, 15 studies met the inclusion criteria and were included in the final analysis (Figure 1) [37–50, 67]. Of note, all eligible studies were case reports/series; no cohort or interventional study met criteria. The full database‐specific search strings and retrieval counts are provided in Appendix S1 (Supporting Information).

### 3.1. Demographic Data and Clinical Profile

Among the 35 reported cases, 22 patients were male (62.8%), while 13 were female (37.2%). The median age at onset was 58 years, ranging from 18 to 80 years. Twenty‐four cases (68.6%) were classified as new‐onset disease temporally following vaccination, while the remaining 11 (31.4%) represented relapses of previously diagnosed lymphomas (Table 1).

**TABLE 1:** Summary of published cutaneous lymphoproliferative disorders temporally associated with SARS‐CoV‐2 (COVID‐19) vaccination (*n* = 35).

**Cases**	**35**	**(%)**

	Gender	Males 62.8
	Age: median (range)	58 years (20–80)

*Non-neoplastic lymphoproliferative disorders*		
	CLH	6 (35.3)
	Lymphomatoid reactions	5 (29.4)
	PCSM‐TCLPD^∗^	5 (29.4)
	PCMZLPD	1 (5.9)
Cutaneous lymphomas	18 (68.6%)	
	LyP	9 (50.0)
	SS	3 (16.7)
	MF	2 (11.2)
	Others	4 (22.1)

*Vaccine types*		
	Pfizer–BioNTech (mRNA)	28 cases (80)
	Moderna (mRNA)	3 cases (8.6)
	AstraZeneca	4 cases (11.4)

*Note:* The table reports demographic characteristics, diagnostic categories/subtypes and vaccine platforms/types for the cases included in this review. CD30‐positive lymphoproliferative disorders include entities within the spectrum of primary cutaneous CD30‐positive T‐cell lymphoproliferative disorders (e.g., LyP and pcALCL) and CD30‐positive large‐cell transformation when specified. PCMZLPD = primary cutaneous marginal zone lymphoproliferative disorder.

Abbreviations: CLH = cutaneous lymphoid hyperplasia, MF = mycosis fungoides, mRNA = messenger RNA, pcALCL = primary cutaneous anaplastic large‐cell lymphoma, SS = Sézary syndrome.

^∗^The case originally reported as PC‐SMPTCL was reclassified as PCSM‐TCLPD according to current WHO–EORTC classification recommendations. LyP, lymphomatoid papulosis; PCSM‐TCLPD, primary cutaneous CD4+ small/medium T‐cell lymphoproliferative disorder.

### 3.2. Diagnostic Classification

Of the 35 cases included, 18 (51.4%) were classified as CLs and 17 (48.6%) as nn‐LPDs. Within the CL group, the most frequently reported entity was LyP (*n* = 9, 50.0%), followed by SS (*n* = 3, 16.7%) and MF (11.2%). The remaining four cases (22.1%) included one each of primary cutaneous γ/δ T‐cell lymphoma (PCGDTCL) and primary cutaneous anaplastic large‐cell lymphoma (pcALCL), and one case described as primary cutaneous CD30+ lymphoproliferative disorder (PCCD30‐LPD), which was not further subclassified. While the latter falls within the CD30+ spectrum, the lack of precise histological distinction between LyP and pcALCL warranted its temporary inclusion in the CL group.

Among cases with available immunophenotyping (*n* = 21), CD30 positivity was detected in 16 (76.2%) and clustered within indolent entities, predominantly LyP (and pcALCL where specified), whereas aggressive CTCLs (e.g., SS and pcGDTCL) were generally CD30‐negative or not reported. This distribution supports the association between indolent clinical course and CD30 expression in our dataset (see Table 2).

**TABLE 2:** Published cases of cutaneous lymphoproliferative disorders temporally associated with SARS‐CoV‐2 (COVID‐19) vaccination.

Title	Authors	Year of publication	Gender	Age	New onset/relapse	Subtype	CD30 +/− or NR	Vaccine	Nr of doses	Time to onset	Clinical manifestation	Therapy	Resolution yes/no	Death? yes/no	Disease‐related death yes/no	CLs or nn‐LPDs
Primary Cutaneous CD4 Small/Medium T‐Cell Lymphoproliferative Disorder Following COVID‐19 Vaccination—What Do We Know About Lymphoproliferative Disorders And Cutaneous Lymphomas After COVID‐19 Vaccination? A Report Of An Atypical Case And A Review Of The Literature	Francisco Javier De La Torre‐Gomar Et Al. [43]	2024	Male	30	New onset	PCSM‐TCLPD	—	BNT162b2®, Pfizer/BioNTech	1	7 days	Nodules and papules	Triamcinolone acetonide intralesional 40 mg/mL	Yes	No	No	nn‐LPDs

Recurrence Of Primary Cutaneous CD30‐Positive Lymphoproliferative Disorder Following COVID‐19 Vaccination	Brumfiel Et Al. [48]	2021	Male	79	Relapse	PCCD30‐LPD	+	BNT162b2®, Pfizer/BioNTech	1	2 days	Ulcerated tumour	Spontaneous regression	Yes	No	No	CLs

Recurrence Of Cutaneous T‐Cell Lymphoma Postviral Vector COVID‐19 Vaccination	Panou Et Al. [47]	2022	Male	60	Relapse	CD30LCT‐MF	+	Chadox1 Ncov‐19	1	28 days	Patches, alopecia, rash and nodules	Not available	Yes	No	No	CLs
			Female	73	Relapse	Lyp Type‐A	+	Chadox1 Ncov‐19	1	10 days	Rash	PUVA	Yes	No	No	CLs

Lymphomatoid Papulosis (Lyp) After AZD1222 And Bnt162b2 COVID‐19 Vaccines	Koumaki Et Al. [40]	2022	Male	60	New onset	Lyp type‐D	+	Chadox1 Ncov‐19	1	7 days	Ulcerated nodule	Spontaneous regression	Yes	No	No	CLs
			Female	66	New onset	Lyp type‐D	+	BNT162b2®, Pfizer/BioNTech	1	10 days	Nodules	NB‐UVB firstly and then methotrexate	No	No	No	CLs

Cutaneous T‐Cell–Rich Lymphoid Infiltrates After SARS‐CoV‐2 Vaccination	Hooper Et Al. [39]	2022	Female	70	New onset	CLH	+	BNT162b2®, Pfizer/BioNTech	1	22 days	Single papule	Surgical excision	Yes	No	No	nn‐LPDs
			Male	50	New onset	CLH	+	BNT162b2®, Pfizer/BioNTech	2	28 days	Single papule	Spontaneous regression	Yes	No	No	nn‐LPDs
			Male	50	New onset	Lyp type‐A	+	BNT162b2®, Pfizer/BioNTech	2	4 days	Papules	Methotrexate	No	No	No	CLs
			Female	20	New onset	Lyp type‐A	+	BNT162b2®, Pfizer/BioNTech	1	42 days	Papules	Methotrexate	No	No	No	CLs

SARS‐CoV‐2 mRNA Vaccine Injection Site Pseudolymphoma	Mintoff Et Al. [49]	2021	Female	68	New onset	CLH	NR	BNT162b2®, Pfizer/BioNTech	2	7 days	Single nodule	Spontaneous regression	Not available	No	No	nn‐LPDs

Real‐World Data On Primary Cutaneous Lymphoproliferative Disorders Following SARS‐CoV‐2 Vaccination: A Multicentre Experience From Tertiary Referral Hospitals	Avallone Et Al. [45]	2022	Female	47	Relapse	CLH	NR	BNT162b2®, Pfizer/BioNTech	2	10 days	Erythematous plaques	Topical corticosteroids	Yes	No	No	nn‐LPDs
			Male	67	Relapse	Lyp type‐A	+	BNT162b2®, Pfizer/BioNTech	4	15 days	Necrotic papules	Methotrexate	Yes	No	No	CLs
			Male	49	Relapse	PCSM‐TCLPD	—	BNT162b2®, Pfizer/BioNTech	4	15 days	Erythematous plaques	Spontaneous regression firstly and then mogamulizumab	Yes	No	No	nn‐LPDs
			Male	58	Relapse	SS	—	BNT162b2®, Pfizer/BioNTech	4	4 days	Erythroderma	Topical corticosteroids	Yes with mogamulizumab	No	No	CLs
			Male	61	Relapse	MF	NR	BNT162b2®, Pfizer/BioNTech	4	14 days	Erythroderma	Oral corticosteroids firstly and then PUVA	Yes with PUVA	No	No	CLs
			Male	61	Relapse	SS	—	BNT162b2®, Pfizer/BioNTech	4	15 days	Suberythroderma	Not available	Not available	No	No	CLs
			Male	55	New onset	CLH	NR	BNT162b2®, Pfizer/BioNTech	4	30 days	Erythematous plaques	Not available	Not available	No	No	nn‐LPDs
			Male	55	New onset	CLH	NR	BNT162b2®, Pfizer/BioNTech	2	7 days	Erythematous plaques	Not available	Not available	No	No	nn‐LPDs
			Female	80	New onset	SS	—	BNT162b2®, Pfizer/BioNTech	4	15 days	Erythroderma	Not available	Not available	No	No	CLs
			Male	60	New onset	Lyp type‐A	+	BNT162b2®, Pfizer/BioNTech	2	30 days	Erythematous papules	Not available	Not available	No	No	nn‐LPDs
			Female	52	New onset	PCSM‐TCLPD	—	BNT162b2®, Pfizer/BioNTech	2	3 days	Erythematous nodule	Radiotherapy	Yes	No	No	nn‐LPDs
			Female	61	New onset	Lyp type‐A	+	BNT162b2®, Pfizer/BioNTech	2	10 days	Erythematous vesicles and pustules	Spontaneous regression	Yes	No	No	CLs
			Male	45	New onset	PCSM‐TCLPD	—	BNT162b2®, Pfizer/BioNTech	4	20 days	Single nodule	Surgical excision	Yes	No	No	nn‐LPDs

Indolent Cutaneous Lymphoma With Gamma/Delta Expression After COVID‐19 Vaccination	Hobayan and Chung [51]	2023	Male	79	New onset	PCGDTCL	—	Moderna COVID‐19 mRNA‐1273	2	3 days	Ulcerated plaque	Doxycycline firstly and then radiotherapy	Yes with radiotherapy	No	No	CLs

Primary Cutaneous Marginal Zone Lymphoproliferative Disorder Following COVID‐19 Vaccination	Stephan Et Al. [44]	2024	Male	57	New onset	PCMZLPD	NR	Moderna COVID‐19 mRNA‐1273	1	14 days	Single nodule	Radiotherapy	Yes	No	No	nn‐LPDs
Cutaneous Lymphoproliferative Disorders After COVID‐19 Vaccination: Clinical Presentation, Histopathology And Outcomes	Gordon Et Al. [38]	2024	Male	18	New onset	Lyp type‐C	+	BNT162b2®, Pfizer/BioNTech	2	30 days	Papules and nodules	Methotrexate	Yes	No	No	CLs
			Male	35	New onset	PC‐SMPTCL	—	BNT162b2®, Pfizer/BioNTech	1	7 days	Nodule	Spontaneous regression	Yes	No	No	CLs

Two Cases Of Challenging Cutaneous Lymphoid Infiltrates Presenting In The Context Of COVID‐19 Vaccination: A Reactive Lymphomatoid Papulosis‐Like Eruption And A Bona Fide Lymphoma	Bresler Et Al. [50]	2023	Male	53	New onset	Lymphomatoid reaction,	+	Moderna COVID‐19 mRNA‐1273	1	7 days	Papules and nodules	Spontaneous regression	Yes	No	No	nn‐LPDs
			Female	62	New onset	Lymphomatoid reaction,	+	BNT162b2®, Pfizer/BioNTech	2	Several	Macules	Spontaneous regression then relapse treated with chemotherapy	No	No	No	nn‐LPDs

Complete Remission Of Primary Cutaneous Follicle Centre Cell Lymphoma Associated With COVID‐19 Vaccine	Aouali Et Al. [37]	2022	Male	63	Relapse	PCBCL	—	Chadox1 Ncov‐19	1	60 days	Nodule	Spontaneous regression	Yes	Not available	Not available	CLs

Primary Cutaneous Anaplastic Large‐Cell Lymphoma With Marked Spontaneous Regression Of Organ Manifestation After SARS‐CoV‐2 Vaccination.	Gambichler Et Al. [42]	2021	Male	57	Relapse	pcALCL	+	BNT162b2®, Pfizer/BioNTech	1	7 days	Nodules	Spontaneous regression	Yes	Not available	Not available	CLs

New Mycosis Fungoides‐Like Lymphomatoid Reaction Following COVID‐19 Vaccination: A Case Report.	Li and Lipson. [41]	2022	Female	56	New onset	Lymphomatoid reaction	NR	BNT162b2®, Pfizer/BioNTech	1	2 days	Patches	Topical corticosteroids	Yes	Not available	Not available	nn‐LPDs

Rare Lymphomatoid Reactions Following SARS‐CoV‐2 Vaccination.	Lewitt Et Al. [46]	2022	Female	54	New onset	Lymphomatoid reaction and PLEVA	+	BNT162b2®, Pfizer/BioNTech	1	7 days	Papules	Doxycycline	Yes	Not available	Not available	nn‐LPDs
			Female	78	New onset	Lymphomatoid reaction	+	BNT162b2®, Pfizer/BioNTech	2	Few	Papule	Not available	Not available	Not available	Not available	nn‐LPDs

*Note:* The table summarizes demographic data, clinical presentation, diagnostic category/subtype, vaccine type (and dose when reported), latency from vaccination to clinical onset/relapse, treatment and outcome. Latency was defined as the interval between vaccine administration and clinical onset/relapse as reported in the original article. When the onset date was not explicitly available, the closest clinically relevant time point provided by the authors (e.g., first clinical assessment or biopsy) was used and specified accordingly. NR, not reported. Percentages and ranges were calculated on available data. PCSM‐TCLPD, primary cutaneous small/medium T‐cell lymphoproliferative disorder; PCCD30‐LPD, primary cutaneous CD30‐positive lymphoproliferative disorder; CD30LCT‐MF, CD30‐positive large‐cell transformation of mycosis fungoides; LyP, lymphomatoid papulosis. PCMZLPD = primary cutaneous marginal zone lymphoproliferative disorder.

Abbreviations: CLH = cutaneous lymphoid hyperplasia, FM = folliculotropic mycosis fungoides, MF = mycosis fungoides, NR = not reported, pcALCL = primary cutaneous anaplastic large‐cell lymphoma, PCBCL = primary cutaneous B‐cell lymphoma, PC‐SMPTCL = primary cutaneous small/medium pleomorphic T‐cell lymphoma, PLEVA = pityriasis lichenoides et varioliformis acuta, SS = Sézary syndrome.

The 17 nn‐LPDs included 6 cases of cutaneous lymphoid hyperplasia (CLH), 5 cases of lymphomatoid reaction (including one MF‐like eruption), 5 cases of primary cutaneous CD4+ small/medium T‐cell lymphoproliferative disorder (PCSM‐TCLPD; 1 case reported as primary cutaneous small/medium pleomorphic T‐cell lymphoma—PC‐SMPTCL—was included within the PCSM‐TCLPD category, in line with the current WHO‐EORTC classification) and 1 case of primary cutaneous marginal zone lymphoproliferative disorder (PCMZLPD) (Table 1).

### 3.3. Vaccination Profile

The majority of patients (*n* = 28, 80.0%) had received the BNT162b2 mRNA vaccine (Pfizer–BioNTech), followed by ChAdOx1 nCoV‐19/AZD1222 (AstraZeneca) in 4 cases (11.4%) and mRNA‐1273 (Moderna) in 3 cases (8.6%) (Table 1). This distribution remained unchanged after reclassifying cases into CLs versus nn‐LPDs, as vaccine exposure was consistent across both neoplastic and non‐neoplastic groups.

### 3.4. Latency to Onset

**FIGURE 2:**
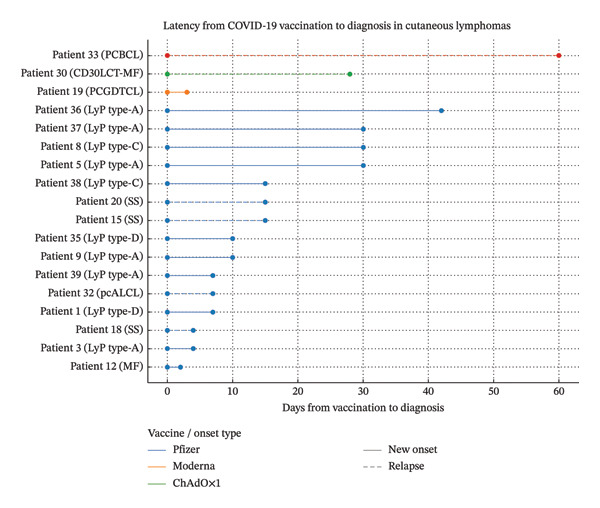
Latency from COVID‐19 vaccination to diagnosis of cutaneous lymphomas (CLs) in 18 reported cases. Each line represents the time interval (in days) between vaccine administration and clinical diagnosis. Solid lines indicate new‐onset CLs, while dashed lines denote relapse of previously diagnosed lymphomas. Line colours correspond to vaccine type: blue for BNT162b2 (Pfizer–BioNTech), orange for mRNA‐1273 (Moderna) and green for ChAdOx1 nCoV‐19/AZD1222 (AstraZeneca). The majority of diagnoses occurred within 14 days of vaccination.

In the subgroup of 17 new‐onset CLs, the time interval between vaccine administration and clinical onset ranged from 3 to 60 days. Most cases occurred within the first 2 weeks following vaccination. Figure 2 provides a graphical representation of time to onset by vaccine type.

## 4. Discussion

The accumulation of temporally associated reports between SARS‐CoV‐2 vaccination and the emergence of CLPDs raises the hypothesis of immune‐mediated triggering events in predisposed individuals. As detailed in Results/Table 2, most reported entities were of T‐cell origin, frequently CD30‐positive and often presented as indolent or self‐limited. Taken together with the group distribution shown in Table 2, this pattern supports the hypothesis that SARS‐CoV‐2 vaccination, particularly via mRNA platforms, may transiently amplify T‐cell activity—possibly unveiling latent lymphoproliferative conditions in immunologically susceptible hosts.

Such findings are consistent with models of vaccine‐associated immune hyperstimulation, wherein activation of effector and memory T cells may uncover previously undiagnosed or quiescent disorders. This effect may be enhanced by the potent activation of dendritic and antigen‐presenting cells induced by mRNA vaccines, promoting T‐cell responses in skin‐resident lymphocyte populations involved in CLs [52].

Among the 35 cases included in this review, most (80%) followed administration of the BNT162b2 vaccine, with smaller proportions linked to ChAdOx1/AZD1222 (11.4%) and mRNA‐1273 (8.6%). These proportions likely reflect overall vaccine availability and administration trends rather than a direct difference in immunogenic potential. Countries with the highest number of published cases also tended to be those with the highest doses administered per 100 inhabitants, as shown by real‐time epidemiological data from global surveillance platforms [53] (see Results). Likewise, the predominance of mRNA vaccines among these reports mirrors the distribution of vaccine types globally: BNT162b2 and mRNA‐1273 were predominantly used in high‐income countries, where most case reports originated, whereas adenoviral vector vaccines, such as ChAdOx1, were more frequently employed in lower‐resource settings. This epidemiological context likely contributes to the association between mRNA vaccines and CTCLs.

Significantly, new‐onset presentations outnumbered reactivations (24/35 vs 11/35; see Results), yet the presence of relapses temporally following vaccination supports the idea of a ‘trigger rather than initiator’ paradigm for a subset of cases. Furthermore, most CTCLs reported were indolent subtypes, particularly LyP, which is associated with favourable prognosis and often presents as a self‐limiting condition. This pattern may reflect the lower immune threshold required to activate or exacerbate these conditions through mechanisms, such as T‐cell hyperactivation and dysregulation of specific cytokine signalling (e.g., IL‐6 and NF‐κB pathways), which are well‐known contributors to lymphoma pathogenesis [54–58].

Latency from vaccination to onset/relapse was short (3–42 days) with clustering in the first two weeks (Results), suggesting a recall‐type immunologic phenomenon, consistent with boosting pre‐existing clonal populations or memory T‐cell responses. [59].

Compared with population‐based CTCL subtype distributions, our case collection shows an apparent over‐representation of indolent entities, many belonging to the primary cutaneous CD30‐positive T‐cell lymphoproliferative disorders. While this could be explained by selective reporting and publication bias, it also raises a hypothesis‐generating possibility that SARS‐CoV‐2 vaccination might act as a trigger in predisposed individuals [34]. In the subset with available immunophenotyping (21/35), 76.2% (16/21) were CD30‐positive [38]. CD30 is a nonspecific activation marker that can be upregulated in T cells in response to viral infections or cytokine surges; and while in these cases it reflects a transient immune activation following immunization, its presence should not be interpreted as evidence of causality or selective involvement of CD30+ clones in postvaccination lymphoproliferative events [60–62].

Preclinical studies have shown that persistent CD30 signalling can activate oncogenic pathways, such as NF‐κB and influence T‐cell differentiation, lending biological plausibility to a transient vaccine‐related effect on CD30+ lymphoproliferative clones. Such mechanisms may transiently affect CD30+ lymphocyte populations, potentially contributing to the observed cases. The indolent nature and high treatment responsiveness of CD30+ disorders likely contribute to the favourable outcomes observed [62]; nevertheless, their over‐representation raises questions about a potentially selective antigen‐driven stimulation following vaccination.

Although causality remains unproven, it is biologically plausible that vaccine‐induced immune activation may interact with pre‐existing but clinically silent lymphoproliferative conditions. Host‐specific factors—whether genetic, epigenetic or related to immune memory—could influence the threshold for disease expression following immunological stimuli. Polymorphisms affecting T‐cell receptor signalling, NF‐κB regulation or cytokine homeostasis may predispose individuals to exaggerated immune responses. Additionally, prior exposure to latent viruses, such as EBV or CMV, may contribute to immune priming and a lowered activation threshold, while speculative, vaccine‐driven epigenetic modulation of immune cell behaviour could also play a role [63, 64].

Notably, vaccine‐associated lymphoproliferative responses are not exclusive to SARS‐CoV‐2 immunization. Similar reports following influenza and HPV vaccines suggest that transient immune stimulation can occasionally unmask subclinical or latent conditions [65]. This phenomenon, referred to as ‘immunologic unveiling’, describes the emergence of overt disease in predisposed individuals following nonspecific immune activation by external stimuli, such as infection or vaccination [66].

Similar mechanisms have been proposed to explain re‐exacerbations of inflammatory dermatoses—including psoriasis, hidradenitis suppurativa and lichen planus—which have been observed to flare or newly arise in temporal proximity to SARS‐CoV‐2 vaccination [18, 19, 67]. These cases further support the notion that systemic immune activation may transiently lower the threshold for disease expression in individuals with underlying immunologic susceptibility.

### 4.1. Study Strengths and Limitations

Study strengths include the detailed extraction of clinical, histopathological and temporal features, as well as the identification of recurring patterns, such as CD30 positivity, indolent course and short latency.

However, the study is limited by its reliance on case reports and small series, which are prone to reporting and publication bias. Moreover, it cannot establish incidence or causality, and therefore over‐represent unusual, temporally striking or clinically indolent presentations while under‐representing common, equivocal or nonreportable events; as a result, signals, such as short‐latency clustering and CD30‐positive/indolent phenotypes, may appear exaggerated relative to their true frequency. The lack of a denominator (vaccinated population at risk) precludes any incidence estimation and inflates the salience of temporal co‐occurrence (‘base‐rate fallacy’), while ascertainment during periods of intense vaccination can further magnify temporal associations. Incomplete follow‐up, heterogeneous diagnostic criteria and lack of molecular data further constrain interpretability and tend to overestimate resolution as well as to underestimate relapses/progression, thus biasing outcomes towards benign trajectories. Under‐reporting of self‐limited or benign events may also skew the clinical spectrum observed. Furthermore, as already highlighted, geographic and vaccine uptake imbalances (predominance of countries with high mRNA use) may skew vaccine distributions towards mRNA platforms, reflecting availability rather than biology. Finally, the absence of molecular/immunogenetic profiling limits causal inference and may mask confounding (e.g., intercurrent infections, booster timing and prior clonal disease), so that any association detected here should be considered hypothesis‐generating rather than evidence of causation.

These limitations underline the intrinsic difficulty of drawing causal inferences in the context of rare diseases based solely on temporally associated reports. While a temporal link has been observed, there is currently no evidence to support a direct pathogenic role of SARS‐CoV‐2 vaccination in triggering CLs. Continued documentation and pharmacovigilance remain essential to better characterize the clinical presentation of these rare events.

## 5. Conclusion

While the benefits of SARS‐CoV‐2 vaccination remain overwhelmingly favourable, this review underscores the importance of maintaining vigilance for rare postvaccination events, including CLs and other nn‐LPDs.

In this contest, we aimed to characterize the onset or relapse of cutaneous lymphoproliferative conditions temporally associated with SARS‐CoV‐2 vaccination. Across 35 cases, we observed a predominance of T‐cell entities, frequent CD30 positivity among indolent phenotypes and a short latency from vaccination to clinical onset/relapse (3–42 days, often within 2 weeks), while the distribution of vaccine platforms mirrored real‐world availability. Taken together, these findings support a hypothesis‐generating signal of transient immune triggering in predisposed hosts, without evidence of causation. Given the case‐level design and reporting biases, incidence cannot be inferred. Clinically, the data support vigilance and careful documentation rather than vaccine hesitancy. Prospective registries with standardized capture of latency, subtype, immunophenotype/molecular data and outcomes are needed to validate these patterns and test mechanistic hypotheses.

Enhanced pharmacovigilance and integration of dermatologic and oncologic registries with vaccine safety databases may improve case recognition and clarify host‐specific vulnerabilities. These observations reinforce—not undermine—the overall safety of COVID‐19 vaccines and should encourage a nuanced, personalized approach to immunization in patients with underlying immunologic or lymphoproliferative conditions.

## Funding

The authors have nothing to report.

Open access publishing facilitated by Universita di Bologna, as part of the Wiley ‐ CRUI‐CARE agreement.

## Conflicts of Interest

The authors declare no conflicts of interest.

## Supporting Information

Additional supporting information can be found online in the Supporting Information section.

## Supporting information


**Supporting Information** Supplementary S1 provides the full database‐specific search strategies, the corresponding retrieval counts, and the supporting data used in this systematic review. These materials support the reproducibility and transparency of the literature search process.

## Data Availability

Data are available upon request from the authors.
