# Supplementary material for: Cutaneous Lymphomas and Lymphoproliferative Disorders Associated With SARS‐CoV‐2 Vaccination: A Systematic Review
Source: J Skin Cancer. 2026 May 28;2026:4893577. doi: 10.1155/jskc/4893577 (PMC13239182; doi:10.1155/jskc/4893577)
Supplement: Supplementary file 1 — Supporting Information Supplementary S1 provides the full database‐specific search strategies, the corresponding retrieval counts, and the supporting data used in this systematic review. These materials support the reproducibility and transparency of the literature search process. [file JSKC-2026-4893577-s001.docx]

**Appendix S1 Full search strategy**

Databases: PubMed/MEDLINE, Scopus, Web of Science Core Collection.
Last search: 20 Mar 2024.
Time coverage: inception–2024.
Export & deduplication: records exported in RIS/CSV and deduplicated in Zotero (key: Author–Year–Title). Duplicates removed: 11.

**PubMed/MEDLINE**

- Search date: 20 Mar 2024
- Query (field tag = Title/Abstract; no limits at search stage):

("cutaneous lymphoma"[Title/Abstract]

OR "cutaneous lymphomas"[Title/Abstract]

OR "mycosis fungoides"[Title/Abstract]

OR "Sézary syndrome"[Title/Abstract]

OR "lymphomatoid papulosis"[Title/Abstract]

OR "CD30"[Title/Abstract]

OR ("primary cutaneous"[Title/Abstract] AND ("T-cell lymphoma"[Title/Abstract] OR "B-cell lymphoma"[Title/Abstract]))

OR "lymphoproliferative disorder*"[Title/Abstract])

AND

("COVID-19 vaccin*"[Title/Abstract] OR "SARS-CoV-2 vaccin*"[Title/Abstract] OR "mRNA vaccin*"[Title/Abstract])

- MeSH secondary search: "Lymphoma, T-Cell, Cutaneous"[MeSH] AND "COVID-19 Vaccines"[MeSH]
- Retrieved: 21

**Scopus** (TITLE-ABS-KEY)

- Search date: 20 Mar 2024
- Query:

TITLE-ABS-KEY

"cutaneous lymphoma" OR "cutaneous lymphomas"

OR "mycosis fungoides" OR "Sézary syndrome"

OR "lymphomatoid papulosis" OR "CD30"

OR ("primary cutaneous" AND ("T-cell lymphoma" OR "B-cell lymphoma"))

OR "lymphoproliferative disorder*")

AND

TITLE-ABS-KEY("COVID-19 vaccin*" OR "SARS-CoV-2 vaccin*" OR "mRNA vaccin*")

- Document types: all (reviews/editorials excluded at screening)
- Retrieved: n = 12

**Web of Science Core Collection** (Topic = Title/Abstract/Keywords)

- Search date: 20 Mar 2024
- Query (TS=):

TS=(“cutaneous lymphoma" OR "cutaneous lymphomas"

OR "mycosis fungoides" OR "Sézary syndrome"

OR "lymphomatoid papulosis" OR "CD30"

OR ("primary cutaneous" AND ("T-cell lymphoma" OR "B-cell lymphoma"))

OR "lymphoproliferative disorder*")

AND TS= ("COVID-19 vaccin*" OR "SARS-CoV-2 vaccin*" OR "mRNA vaccin*")

- Indexes/Timespan: [es. SCI-EXPANDED, SSCI; all years]
- Retrieved: n = 10

Limits applied after full-text screening

- Language: English
- Population: ≥18 years
- Diagnosis: histopathology-confirmed cutaneous lymphoma
- Study design: case reports/series, observational studies, trials (reviews/editorials excluded)

**Inizio modulo**

**Fine modulo**
